# Enhanced Chromium (VI) Adsorption onto Waste Pomegranate-Peel-Derived Biochar for Wastewater Treatment: Performance and Mechanism

**DOI:** 10.3390/toxics11050440

**Published:** 2023-05-07

**Authors:** Yingzhou Chen, Jinyan Yang, Adil Abbas

**Affiliations:** College of Architecture and Environment, Sichuan University, Chengdu 610065, China; yingzhouchen@stu.scu.edu.cn (Y.C.); yanyang@scu.edu.cn (J.Y.)

**Keywords:** pomegranate peel, biochar, chromium, adsorption mechanism, water purification

## Abstract

Surface chemical modification allows for the rational construction of biochar with desirable structures and functionalities for environment purification. Fruit-peel-derived adsorbing material has been well studied in the adsorption of heavy-metal removal due to its abundance and non-toxicity, but its precise mechanism in removing chromium-containing pollutants remains unclear. Herein, we explored the potential application of engineered biochar prepared from fruit waste via chemical modification to remove chromium (Cr) from an aqueous solution. By synthesizing two types of agricultural residue-derived adsorbents, including pomegranate peel adsorbent (PG) and its modified product, pomegranate-peel-derived biochar (PG-B), via chemical and thermal decomposition methods, we elucidated the adsorption property of Cr(VI) on the studied materials and identified the cation retention mechanism of the adsorption process. Batch experiments and varied characterizations demonstrated that superior activity was exhibited in PG-B, which can contribute to the porous surfaces caused by pyrolysis and effective active sites resulting from alkalization. The highest Cr(VI) adsorption capacity is obtained at pH 4, a dosage of 6.25 g L^−1^, and a contact time of 30 min. The maximum adsorption efficiency of 90.50% in a short period (30 min) was obtained on PG-B, while PG reached a removal performance of 78.01% at 60 min. The results from kinetic and isotherm models suggested that monolayer chemisorption dominated the adsorption process. The Langmuir maximum adsorption capacity is 16.23 mg g^−1^. This study shortened the adsorption equilibrium time of pomegranate-based biosorbents and presents positive significance in designing and optimizing waste fruit-peel-derived adsorption materials for water purification.

## 1. Introduction

Chromium (Cr), one of the typical toxic heavy metal ions with the common forms of trivalent chromium (Cr(III)) and hexavalent chromium (Cr(VI)) in the soil–liquid nexus, has induced increased pollution in water sources and severe neurotoxicity to humans, posing a prominent concern among varied environmental issues [1,2,3]. Much of the excess chromium accumulating in the natural environment comes from human industrial activities (such as leather, electroplating, printing, dyeing, etc.) and has been proven to be poisonous to vital organs and systems of the human body [4,5,6,7,8]. Furthermore, Cr(VI) has more substantial solubility than Cr(III) and can quickly enter human cells, cause damage to the liver, kidney, and other internal organs, and accumulate in the human body [9]. Worse yet, Cr(VI) is carcinogenic and may induce gene mutations [10]. The maximum concentration of Cr(VI) in surface water (0.1 mg L^−1^) has been restricted by the World Health Organization (WHO) [11,12]. Hence, numerous studies have been conducted to mitigate Cr(VI) poisoning and center the environmental remediation of polluted soil and water body, emphasizing the significant necessity for developing practical approaches to get rid of Cr(VI) from wastewater [13,14].

Adsorption of Cr(VI) by eco-friendly and economical adsorbents is among the most promising methods for water treatment, which has spurred many studies due to its simple operation, no secondary pollution, high efficiency, low price, and reusable characteristics, preventing plenty of shortcomings in other methods, such as high cost, large energy consumption, membrane pollution, intricate operation process, and secondary toxic sludge pollution that caused limitations in the removal efficiency of Cr(VI) [15,16,17]. Biochar is a carbonaceous solid residue obtained from the pyrolysis and carbonization of readily available carbon-rich biomass, such as agricultural waste, forest residues, wood, algae, animal waste, activated sludge, etc., which has been extensively investigated as a promising sorbent for removing heavy metals due to its abundant oxygen-containing functional groups, large specific surface area, disordered structures, and a high degree of aromatic structure. The structural characteristics and sorption properties of biochar produced from varied biowaste have been widely studied [18]. Biochar can remove heavy metals from the environment primarily through physical adsorption, ion exchange, electrostatic interaction, and complexation [19,20,21]. Its physical and chemical properties can be optimized according to the characteristics of different pollutants, including exogenous media modification (metal negative magnetic, acid-base oxidation, coating, and impregnation) and carbonization technology (gas activation, microwave carbonization, and ball milling modification) [22,23]. Recently, biochar adsorbents derived from fruit waste such as orange peel [24], grapefruit peel [25], apple peel [26], sweet lime peel [27], durian peel [28], and pomelo peel [29] have been proven to effectively remove heavy metal from polluted soil and aqueous solutions. For instance, Basu, Guha, and Ray [19] applied modified cucumber peel as a novel adsorbent to remove lead (Pb) in water samples, where a completed adsorption process was observed within 60 min and the important role of the carboxyl group in metal binding was confirmed.

Pomegranate (*Punica granatum* L.), a common fruit grown in temperate and tropical regions, possesses an essential value for development and utilization in food, medicine, and cosmetics. Pomegranate peel, as a byproduct of varied fruit processing industries, makes up approximately 40~50% of the total weight of pomegranate and often induces environmental pollution and resource waste [30,31,32]. Pomegranate peel is rich in functional groups such as carboxylic, hydroxyl, and lactone groups due to its high content of polymers that include cellulose, hemicellulose, and lignin, which is beneficial to selective adsorption of heavy metal ions [33]. More importantly, chemical modification and thermal decomposition of waste biomass can effectively increase the number of active sites and improve the adsorption affinity between materials and heavy-metal pollutants. The structural characteristics and potential applications of peel-derived adsorption materials have been widely studied, while few studies focused on the effects of chemical modification and pyrolysis treatment on the physicochemical properties of peel adsorption materials, including material surface properties and adsorption characteristics of heavy metal ions [34,35]. Furthermore, the application advantages of biochar materials in the treatment of heavy-metal pollution remain unclear.

In this work, we developed two types of agricultural residue-derived adsorbent materials, including pomegranate peel adsorbent (PG) and its modified product, pomegranate-peel-derived biochar (PG-B), by using pomegranate peel as the raw material and explored the difference in adsorption characteristics of Cr(VI) species on the studied materials. Batch experiments demonstrated the important role that environmental factors played in the removal performance of Cr(VI) on PG and PG-B, including the solution pH, adsorbents dosage, contact time, and initial Cr(VI) concentration. Disparities in the removal efficiency and underlying adsorption mechanism were disclosed based on the experimental and characterization analysis. This study provides a new strategy for constructing promising adsorbents by adopting cheap, readily available biomass materials to efficiently eliminate heavy-metal pollutants.

## 2. Materials and Methods

### 2.1. Materials

Sodium hydroxide (NaOH), nitrosylsulfuric acid (NOHSO_4_), and sodium pyrosulfite (Na_2_S_2_O_5_) were purchased from Chengdu Ke Long Chemical Industry Co., Ltd. (Chengdu, China). The pomegranate peels used as the starting materials were collected from Sichuan province, China (30°36′ N, 103°59′ E). All chemicals and reagents used in this study were analytical grade. Deionized water was used for all synthesis and batch experiments.

### 2.2. Preparation of PG and PG-B

The collected pomegranate peels were cleaned with deionized water and kept in the air blast drying oven at 80 °C for 15 h, followed by cooling to room temperature in a drying vessel. Subsequently, the obtained products were sieved to 0.3 mm followed by pouring them into 0.5 M of NaOH aqueous solution and keeping them for 5 h at room temperature. After that, the mixed solution was dried at 80 °C for 24 h. Finally, the PG adsorbent was obtained and kept in a drying vessel for further use after cooling down to room temperature.

To prepare PG-B adsorbents, the screened pomegranate peels were soaked in NOHSO_4_ solution for 24 h followed by being filtered and cleaned with deionized water. Then, the obtained material was transferred to a muffle furnace with heating to 600 °C at a heating rate of 5 °C min^−1^. After being calcined for 2 h and naturally cooled down to room temperature, the products were kept in 0.5 M of Na_2_S_2_O_5_ aqueous solution for 24 h. After being filtered and washed with deionized water to remove excess surface ions, the obtained products were dried at 80 °C for 5 h.

### 2.3. Adsorption Experiments

Batch experiments were conducted to explore the adsorption properties of PG and PGB for Cr(VI)removal under a set of reaction parameters including different contact times (10, 20, 30, 40, 50, 60, 70, 80, 90, and 100 min), solution pH (4, 6, 7, 8, and 10), material doses (2.50, 3.75, 5.00, 6.25, and 7.50 g L^−1^), and initial Cr(VI) concentrations (10, 20, 30, 40, and 50 mg L^−1^). For each experiment, the reaction mixture containing 20 mL of Cr(VI) aqueous solution with the desired concentration and adsorbents in specific dosages was equilibrated in a 50 mL polyethylene centrifuge tube and then put on the thermostatic oscillator operating at 150 rpm and 25 °C. After the reaction, the mixture was filtered through 0.45 μm filters, and the obtained supernatant was used to determine the Cr(VI) content following the 1,5-diphenylcarazide colorimetric method using a UV−Vis spectrophotometer (Thermo Fisher, Genesys180, Waltham, MA, USA). The Cr(VI) adsorption capacity (Q_e_) and percentage removal efficiency were calculated as Equations (1) and (2), respectively.

(1) $$Q_{e}=\frac{\left(C_{0}-C_{e}\right)V}{m}$$

(2) $$\mathrm{Removal}\;\mathrm{efficiency}\;\left(\%\right)=\frac{\left(C_{0}-C_{e}\right)\times 100}{C_{0}}$$

where C_0_ and C_e_ stand for the Cr(VI) concentration at the initial and adsorption equilibrium (mg L^−1^), respectively. V denotes the volume of the experimental solution (L) and m represents the weight of the adsorbents (g).

### 2.4. Kinetics and Isotherm Analysis

To elucidate the adsorption mechanism of Cr(VI) on the studied adsorbents (PG and PG-B), typical kinetic models and isotherm models were applied to simulate the obtained experimental data. The adsorption kinetics was described by the pseudo-first-order (PFO), pseudo-second-order (PSO), and Elovich models according to Equations (3)–(5), while the Langmuir, Freundlich, and Dubinin–Radushkevich (D-R) models were used to fit the experimental data of equilibrium adsorption of Cr(VI) by PG and PG-B according to Equations (5)–(7), respectively.

(3) $$\mathrm{Pseudo}-\mathrm{first}-\mathrm{order}\;(\mathrm{PFO}):\;\ln\left[Q_{e}-Q_{t}\right]{=\mathrm{lnQ}}_{e}-k_{1}t$$

(4) $$\mathrm{Pseudo}-\mathrm{second}-\mathrm{order}\;(\mathrm{PSO}):\;\frac{t}{Q_{t}}=\frac{1}{K_{2}Q_{e}^{2}}+\frac{t}{\;\mathrm{Qe}}$$

(5) $$\mathrm{Elovich}\;\mathrm{model}:\;Q_{t}=\frac{1}{b}\ln\left(t\right)+\frac{1}{b}\ln(\mathrm{ab})$$

(6) $$\mathrm{Langmuir}\;\mathrm{model}:\;Q_{e}=\frac{Q_{0}Q_{e}K_{L}}{\left({1+C}_{e}K_{L}\right)}$$

(7) $$\mathrm{Freundlich}\;\mathrm{model}:\;Q_{e}{=K}_{F}C_{e}^{1/n}$$

(8) $$\mathrm{Dubinin}-\mathrm{Radushkevich}\;(D-R)\;\mathrm{model}:\;{\mathrm{lnQ}}_{e}{=\mathrm{lnQ}}_{0}-{\beta \varepsilon }^{2}$$

$$\varepsilon =\mathrm{RTln}(1+\frac{1}{C_{e}})$$

$$E=\frac{1}{{\left(2\beta \right)}^{0.5}}$$

where C_e_ (mg L^−1^) denotes the equilibrium concentration of adsorbates; Q_t_ and Q_e_ (mg g^−1^) stands for the amounts of Cr(VI) adsorbed at time t (min) and at equilibrium; Q_0_ (mg g^−1^) is the maximum adsorption capacity of adsorbents; K_F_ [(mg g^−1^)(L mg^−1^)^1/n^] and K_L_ (L mg^−1^) are the Freundlich and Langmuir equilibrium constants, respectively; and 1/n is the adsorption strength. k_1_ (min^−1^) and k_2_ (g mg^−1^ min^−1^) stand for the adsorption rate constants of the pseudo-first-order and pseudo-second-order models, respectively. a (mg (g h)^−1^) and b (g mg^−1^) denote the initial rate constant and desorption constant of the Elovich model. β represents the constant of the D-R model; ε stands for Polanyi adsorption potential; R is the ideal gas constant of 8.314 J (mol K)^−1^; T denotes the absolute temperature; E is the adsorption-free energy (J mol^−1^).

### 2.5. Characterization of Materials

The surface structure and element changes of PG and PG-B were analyzed by a scanning electron microscope equipped with an energy-dispersive spectrometer (SEM-EDS, Prox, Phenom, The Netherlands). The Fourier transform infrared (FT-IR) spectra were recorded in the 4000–400 cm^−1^ region by an infrared spectrophotometer (Thermo Nicolet, MA, Madison, WI, USA).

## 3. Results and Discussion

### 3.1. Characterization of PG and PG-B

**Figure 1:**
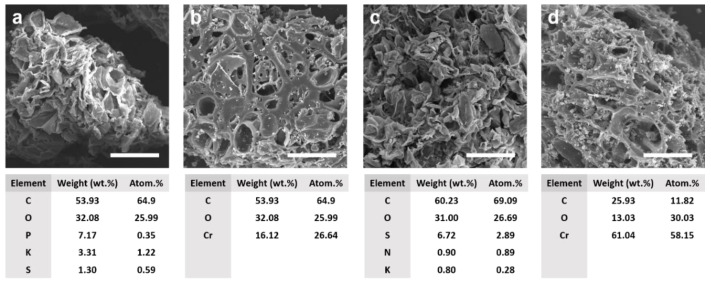
SEM images of (**a**) PG, (**b**) PG after adsorption reaction, (**c**) PG-B, and (**d**) PG-B after adsorption reaction with corresponding element content distributions.

The morphology and element analysis of the pomegranate peel adsorbent (PG) obtained by alkali treatment and pomegranate-peel-derived biochar (PG-B) prepared by pyrolysis and acid treatment has been described in Figure 1 via the SEM-EDS spectra, which indicated that chemical modification and pyrolysis treatment significantly affected surface structures of the pomegranate-peel-derived adsorption materials. As compared to PG adsorbents, more complicated microporous and loose structures were obtained on PG-B adsorbents, which may be attributed to the fact that high-temperature calcination and subsequent strong acid treatment increased the pore and specific surface area of the materials [20]. The surface of PG-B was heterogeneous, porous, and honeycombed, while the surface of the raw material was disordered, rough, and tightly bonded. The honeycomb structure formed by the planar arrangement of multiple layers of carbon increased the specific surface area of materials, leading to enhanced removal performance of Cr(VI) [36]. EDS analysis results showed that C and O, which were 64.9% and 25.99% in PG and 69.09% and 26.69% in PG-B, respectively, were the main elements of the two modified materials derived from pomegranate peels. After pyrolysis and chemical modification by NOHSO_4_ and Na_2_S_2_O_5_, PG-B adsorbents showed markedly increased S and N content while P and K were the main characteristic element of PG adsorbents, which can be ascribed to the high temperature that affected surface properties of PG-B adsorbents. After the adsorption reaction, a much higher Cr content of 58.15% in PG-B than in PG adsorbents with a Cr content of 26.64% was observed, indicating that pomegranate-peel-derived biochar exhibited enhanced adsorption capacity.

**Figure 2:**
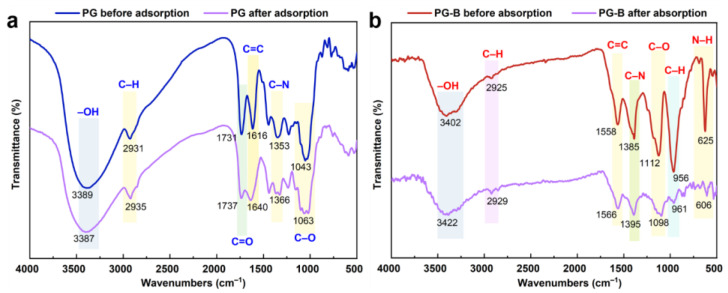
Fourier-transform infrared spectra (FT-IR) spectra of (**a**) PG and (**b**) PG-B.

Functional groups on PG and PG-B were elucidated by the FT-IR spectra (Figure 2). The variations in the wave number of the main peaks associated with the studied materials before and after the adsorption reaction are presented in Table 1. The spectra of both PG and PG-B showed a strong and wide adsorption peak around 3000–3500 cm^−1^, which can be assigned to the stretching vibrations of −OH existing in carboxylic acids, phenols, or alcohols [37]. The peaks that occurred near 2931 cm^−1^ of PG and 2914 cm^−1^ of PG-B were caused by the stretching vibration of the structural C–H of aliphatic methylene groups, where a significant diminution in intensity was observed in PG-B [38,39]. The ester–C=O band at 1731 cm^−1^ was observed in the spectrum of PG but was absent in PG-B. Moreover, the C−O at 1043 cm^−1^ moved to a high wavenumber at 1122 cm^−1^. The above results implied that oxidation of the pomegranate peel was influenced markedly by thermal decomposition and chemical modification by NOHSO_4_ and Na_2_S_2_O_5_. After the adsorption of Cr(VI), redshift and attenuation of the peak intensity were observed on both PG and PG-B, for instance, the band at 1731 cm^−1^ in the spectrum of PG adsorbents moved to 1737 cm^−1^ as the peak intensity also decreased; for the spectrum of PG-B, the band at 1560 cm^−1^ moved to 1566 cm^−1^ and 1383 cm^−1^ moved to 1396 cm^−1^ with the peaks showing a decline in intensity, demonstrating that the surface functional groups on the material’s surface, such as –OH, C=O, and –NH_2_, were responsible for Cr(VI) adsorption. After the Cr(VI) adsorption on PG-B, the peak at 625 cm^−1^ resulting from the bending of N–H bonds showed a significant decline in intensity, indicating that superficial –NH_2_ was an important contributor to Cr(VI) removal [40,41]. These results indicated that both PG and PG-B were rich in oxygenated functional groups (–OH, C–H, C–N, –NH_2_, and C–C) while PG-B had more abundant functional groups, which had great potential for the adhesion of Cr(VI).

### 3.2. Effect of pH on the Adsorption Process

**Figure 3:**
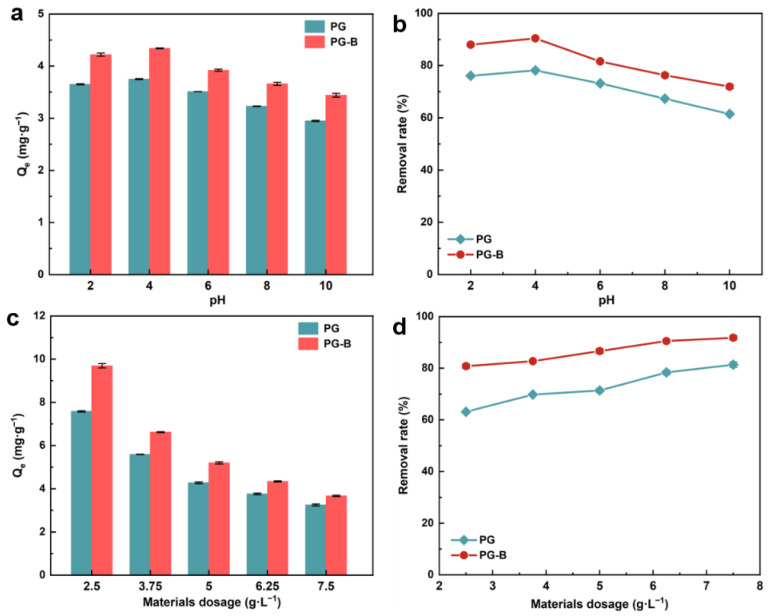
Effect of solution pH on (**a**) Cr(VI) adsorption capacity and (**b**) removal efficiency by PG and PG-B. Condition: Initial Cr(VI) concentration = 30 mg L^−1^, adsorbent dosages = 6.25 g L^−1^, reaction time = 60 min. (**c**) Cr(VI) adsorption capacity and (**d**) removal efficiency by PG and PG-B at different materials dosage. Condition: Initial Cr(VI) concentration = 30 mg L^−1^, pH = 4, reaction time = 60 min.

The influence of solution pH on Cr(VI) adsorption by PG and PG-B was explored, and strong pH dependence is presented in Figure 3a,b. The solution pH value, as one of the essential parameters, can not only affect the charge and ion conditions of the material surface but also dominate the Cr(VI) form in solution, causing the difference in chemical behavior of Cr(VI) such as coordination, complexation, and electrostatic interaction in the system with a changed pH value [42,43]. Specifically, the hydrolysis of Cr(VI) primarily produced CrO_4_^2−^, Cr_2_O_7_^2−^, and HCrO_4_^2−^, where the hydrolysis product was largely HCrO_4_^2−^ and Cr_2_O_7_^2−^ at pH 2~6 while CrO_4_^2−^ dominated when pH > 6 [44]. Similar responses to the pH variation and changes in the adsorption performance showing a slow increase in adsorption capacity followed by a rapid decrease with a rising pH were observed in the studied reaction system with PG and PG-B, indicating that the interaction between chromium and the surface of pomegranate-peel-derived adsorbents was strongly affected by the solution pH value. The maximum adsorption capacity of both PG and PG-B was reached at pH = 4, where the adsorption amount of Cr(VI) on PG-B and PG reached 4.34 mg g^−1^ and 3.75 mg g^−1^ with the corresponding removal efficiency of 90.41% and 78.17%, respectively, while a significant decline in removal efficiency was presented in other pH conditions. When pH > 4, the adsorption capacity of Cr(VI) on both P PG and PG-B decreased rapidly with the increase in pH value. For PG-B, the adsorption capacity decreased from 4.34 mg g^−1^ to 3.44 mg g^−1^ as the corresponding removal efficiency decreased from 90.41% to 71.91%, while the adsorption capacity decreased from 3.75 mg g^−1^ to 2.95 mg g^−1^ as the corresponding removal efficiency decreased from 78.17% to 61.46%. This may be attributed to the enhanced degree of protonation of the amine group and other surface functional groups on the adsorbents in favor of the electrostatic attraction to the chromium-containing anions in reaction systems, resulting in the superior performance of pomegranate-peel-derived biochar PG-B rich in –NH_2_ under low pH conditions. Moreover, another pathway of enhanced adsorption of Cr(VI) in the acid reaction system was the strong reducing action of electron donor groups (such as –OH and –COOH), causing the conversion of Cr(VI) to Cr(III) [45]. The superficial accumulation of negative charge resulting from the increased pH value and decreased Cr(VI) adsorption quantity caused the transformation of the main existence form of Cr(VI) into CrO_4_^2−^, which could be attributed to poor removal efficiency [46]. The above results elucidated that PG and PG-B were most effective for Cr(VI) removal at pH = 4, which agree with previous studies presenting similar tendencies that the maximum adsorption capacity was obtained under acidic conditions (pH = 3~5) [30,39].

### 3.3. Effect of Material Dosage on the Adsorption Process

The role of adsorbent dosages on the Cr(VI) removal efficiency of PG and PG-B was investigated (Figure 3c,d). It was observed that the increased addition of adsorbents was beneficial to Cr(VI) adsorption efficiency on both PG and PG-B. Specifically, the removal efficiency of Cr(VI) on PG and PG-B increased from 63.13% to 81.35% and from 80.77% to 91.81%, respectively, while the corresponding adsorption capacity of Cr(VI) decreased from 7.58 mg g^−1^ to 3.25 mg g^−1^ and from 9.69 mg g^−1^ to 3.67 mg g^−1^ with an increase in the material dosage from 2.5 to 7.5 g. In addition, a slow rise in the removal efficiency followed by reaching stabilization at the adsorbent dosage of 6.25 g L^−1^ was observed in the reaction system with PG-B while that of PG was on the constant rise though Cr(VI) adsorption efficiency of PG-B was always greater than that of PG, which indicated that increasing additions of adsorbents affected PG more than PG-B. The above-mentioned dosage-dominated efficiency promotion can be ascribed to the higher specific surface areas and more active sites, which was beneficial to Cr(VI) adsorption in the reaction system [47]. These results indicated that increasing the adsorbent addition can facilitate the removal of Cr(VI) by providing more active sites. In consideration of the removal efficiency and material cost, an addition of 6.25 g L^−1^ was suitable as the optimal dosage in the latter experiments.

### 3.4. Effect of Contact Time

**Figure 4:**
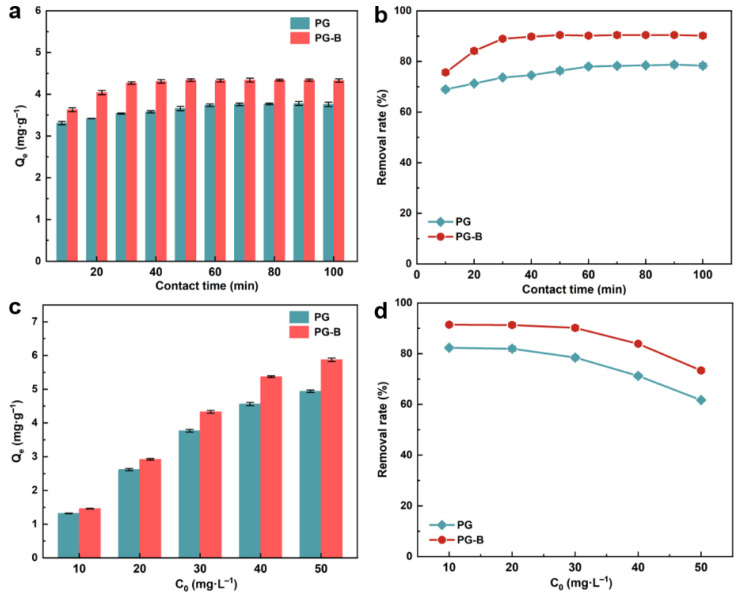
Effect of contact time on (**a**) Cr(VI) adsorption capacity and (**b**) removal efficiency by PG and PG-B. Conditions: Initial Cr(VI) concentration = 30 mg L^−1^, adsorbents dosage = 6.25 g L^−1^, pH = 4. (**c**) Cr(VI) adsorption capacity and (**d**) removal efficiency by PG and PG-B at different initial concentrations of Cr(VI). Condition: Adsorbents dosage = 6.25 g L^−1^, pH = 4, reaction time = 60 min.

The removal efficiency of Cr(VI) in the studied reaction systems with PG and PG-B increased within 0 to 100 min (Figure 4b), where the adsorption equilibrium of both adsorbents was reached in a relatively short time, indicating the strong interaction between materials and Cr(VI). A significant increase in Cr(VI) adsorption efficiency on PG-B was presented at the initial 30 min with nearly 89% Cr(VI) removed, followed by reaching the stable state after 40 min of the reaction while the adsorption equilibrium of Cr(VI) on PG was reached at 60 min of reaction. The reaction solution treated with PG-B showed the highest adsorption capacity of 4.34 mg g^−1^, presenting the highest removal efficiency of 90.41%. The rapid enhancement of the removal rate of Cr(VI) on PG and PG-B in the primary stage can be attributed to the abundant unused superficial adsorption sites. As the reaction progressed, the slowdown in the adsorption reaction was presented until the adsorption equilibrium was reached, which may be due to the fact that the binding sites were occupied by the oxygen anion containing chromium and interference between the excess adsorbents, therefore the active sites decreased [48]. We can conclude that the optimal reaction time for removing Cr(VI) by PG and PG-B was 60 min and 30 min, respectively. Similar diurnal variation trends of the removal rate were presented for Cr(VI) on pine cones gel beads nanocomposite modified by ferroferric oxide and on pomegranate-derived adsorbents [49,50].

### 3.5. Effect of Initial Concentration

The adsorption performance in varied initial Cr(VI) concentrations was investigated (Figure 4c,d). As expected, the adsorption capacity of Cr(VI) on PG and PG-B presented a significant promotion with an increasing Cr(VI) concentration of the starting solution, which was in accordance with previous studies reporting that high initial metal concentrations in the reaction system could conduct the rapid mass transfer of pollutants and readily saturate binding sites on the surface of adsorbents [51,52,53]. Furthermore, when the initial Cr(VI) concentration went up to 50 mg L^−1^, PG-B with a maximum adsorption capacity of 5.87 mg g^−1^ performed significantly better than PG, which reached 4.94 mg g^−1^ at the adsorption equilibrium. As compared to PG, enhancing the initial Cr(VI) concentration showed a greater influence on the adsorption performance of PG-B with the adsorption capacity maintaining a steady increase, indicating the slower saturation of adsorption sites on the PG-B surface. However, the adsorption efficiency of Cr(VI) on PG and PG-B decreased from 82.34% to 61.72% and 91.42% to 73.39%, respectively, upon increasing the Cr(VI) concentration from 10 mg g^−1^ to 50 mg g^−1^, which may be attributed to the fact that the limitation in effective binding sites on the fixed number of PG and PG-B particles in the solution hindered the Cr(VI) removal. Considering the practical application and removal performance, 30 mg L^−1^ was the applicable concentration for further experiments.

### 3.6. Mechanism Study

#### 3.6.1. Adsorption Isotherms

**Figure 5:**
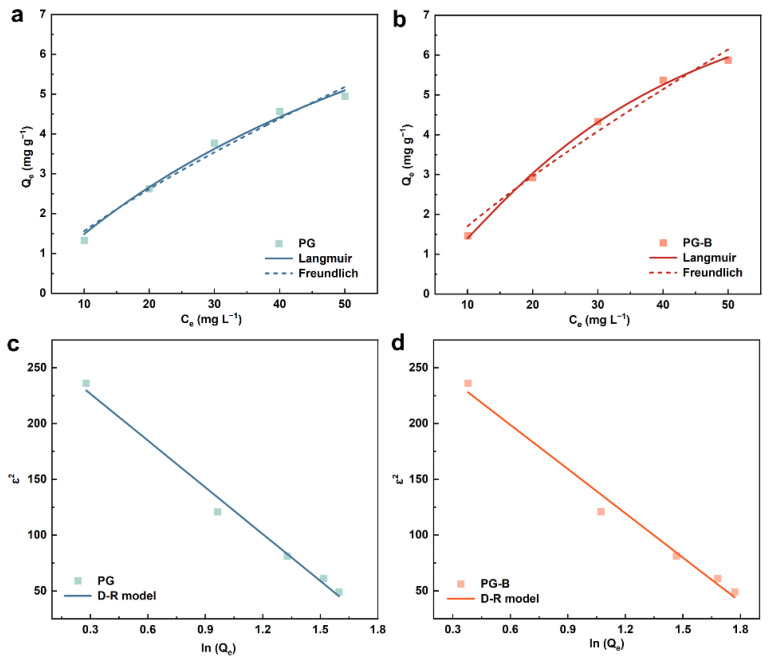
Linearized Langmuir and Freundlich plots for the adsorption of Cr(VI) on (**a**) PG and (**b**) PG-B; D-R model for Cr(VI) adsorption on (**c**) PG and (**d**) PG-B.

The adsorption isotherm played a crucial role in studying the adsorption process, indicating the strength of the interaction between adsorption materials and pollutants. The adsorption state of pollutants on the interface of adsorption materials and the structure of the adsorption layer can also be obtained by analyzing the shape and change of the adsorption isotherm. In this study, the Langmuir, Freundlich, and D-R models were used to fit the data obtained from the adsorption equilibrium (Figure 5). According to the fitting parameters presented in Table 2, both the Freundlich and Langmuir models were appropriate for the Cr(VI) adsorption on PG and PG-B, while the latter presented a higher fitting degree, indicating that Cr(VI) adsorption on pomegranate-peel-derived adsorbents (PG and PG-B) was monolayer adsorption, where homogeneity adsorption sites and the binding process influenced by the chemical–physical interaction were involved [54,55]. According to the relevant results of the Langmuir model, the theoretical adsorption capacity of PG-B (16.229 mg g^−1^) was 26.9% higher than that of PG (12.793 mg g^−1^). The maximum adsorption capacity of the adsorbents in this study and of different bio-waste adsorbents used for the adsorption of water contaminants recently described in the literature was reported for various bio-waste adsorbents on pollutants and is summarized in Table 3. It is evident that PG-B has a stronger adsorption capacity for Cr(VI) of PG-B as it was considerably higher than the other bio-waste adsorbents, proving the superior performance of PG-B adsorbent due to its structural property. Compared with the previous pomegranate peel adsorbent, the PG-B adsorbent in this study achieved the adsorption equilibrium in a shorter time, showing great potential in the effective removal of adsorbents for Cr(VI) adsorption in wastewater purification. The adsorption equilibrium constants (R_L_) of the two reaction systems obtained by further calculation (*R*_L_ = 1/(1 + a × C_0_) were located at 0~1, confirming beneficial adsorption. In addition, good linearity of the D-R model was found for Cr(VI) adsorption on both PG and PG-B with correlation coefficients (R^2^) greater than 0.9 and average adsorption energy (E) between 8 kJ·mol^−1^ and 16 kJ·mol^−1^, indicating that chemical adsorption played a leading role in the adsorption process of Cr(VI) on PG and PG-B.

#### 3.6.2. Adsorption Kinetics

**Figure 6:**
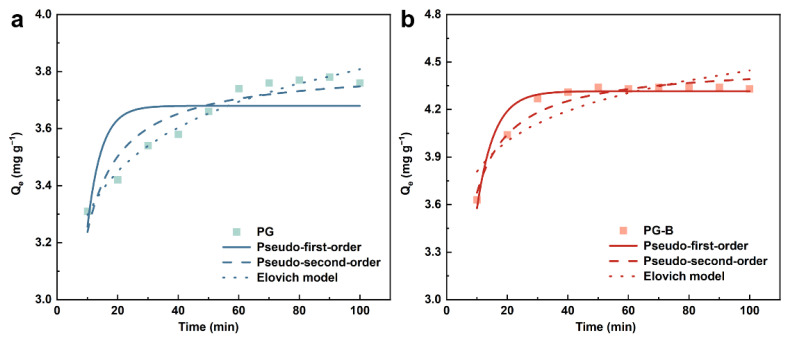
Pseudo-first-order (solid lines), pseudo-second-order (dashed lines), and Elovich models (dotted lines) for Cr(VI) adsorption on (**a**) PG and (**b**) PG-B.

In consideration of the adsorption rate, one of the critical parameters determining adsorption performance, a slow adsorption process of Cr(VI) was observed in the reaction system treated with PG while the Cr(VI) removal by PG-B increased rapidly at first followed by slow adsorption. On the one hand, a large number of binding sites contributed to the rapid adsorption during the initial stage of the reaction. On the other hand, efficient mass transfer resulted from the concentration difference between the surface of the adsorbents and the solution, which facilitated the combination of Cr(VI) and unoccupied active sites. This faster adsorption rate has apparent advantages in practical applications. To gain further insight into the removal performance and adsorption behavior characteristics of Cr(VI) on pomegranate-peel-derived adsorbents-, several typical kinetic models, including the pseudo-first-order model, the pseudo-second-order model, and the Elovich model, were employed to evaluate the dynamics of the adsorption process of Cr(VI) on the studied adsorbents. The fitting results of the above kinetic models are shown in Figure 6, with the parameters of the kinetics equations exhibited in Table 2. For PG adsorbents, the equilibrium concentration calculated by the pseudo-first-order model differed greatly from the experimental value, and its R^2^ was relatively small, indicating that the pseudo-first-order model was not suitable for the adsorption of Cr(VI) by PG. From the parameters listed in Table 4, a correlation coefficient higher than 0.9 suggested that the adsorption of Cr(VI) onto the PG adsorbent followed the Elovich model, indicating that PG has uniformly distributed surface adsorption energy during the entire adsorption process. As shown in Table 4, the pseudo-second-order model, which emphasized chemisorption as the rate-limiting step, can describe the kinetics of Cr(VI) by PG and PG-B with high correlation coefficient values (R^2^ > 0.9). In addition, the theoretical adsorption capacities of Cr(VI) onto PG (3.813 mg g^−1^) and PG-B (4.489 mg g^−1^) were in better agreement with the experimental data (3.76 mg g^−1^ for PG and 4.34 mg g^−1^ for PG-B). A similar chemisorption-dominated removal mechanism was presented for Cr(VI) on wheat-residue-derived black carbon [65], sunflower waste carbonaceous adsorbents [66], agricultural waste, and timber industry waste carbons [67], in which reduction, ion exchange, and complexation were also involved [68,69]. In summary, the adsorption process of Cr(VI) onto PG and PG-B adsorbents can be ascribed to the result of a combination of multiple mechanisms due to the complex chemical composition of peel-derived materials rich in binding sites for heavy metals.

Based on the above isotherms and kinetics analysis, the Cr(VI) adsorption process on PG and PG-B was dominated by chemical adsorption and single-layer adsorption. The enhanced adsorption reaction at low pH could be induced by the electrostatic attraction between the protonated surface of materials and the oxygen-containing anion of chromium. Regarding data obtained from FT-IR analysis, the variation peaks after the adsorption reaction, which can be ascribed to oxygen-containing functional groups (such as phenolic hydroxyl, alkoxy, and carboxyl groups), were involved in PG and PG-B adsorbents, which were in accordance with the fact that pomegranate peel contained abundant flavonoids rich in C=C and C=O bonds [70], alkaloids with nitrogen-containing organic compounds [71], gallic acid, and phenols, which contained large amounts of –OH [72,73]. The above results showed that the bond energy interaction and electrostatic attraction between adsorbents and adsorbates could aptly describe the removal process of Cr(VI) on PG and PG-B, which can be summarized as the following three aspects: (i) The electrostatic interaction between Cr(VI) and protonated amine groups on adsorbents surface [74]; (ii) the reduction of hexavalent Cr(VI) to Cr(Ⅲ) by substantial electron donors on the material surface, including C–C, C–H, –OH, and –COOH [33]; and (iii) the complexation between metal ions and oxygen-containing surface functional groups, such as –COOH, –OH, and –ROH [75].

## 4. Conclusions

In summary, the application of agricultural residue-derived adsorbents, including pomegranate peel adsorbent (PG) and its modified product, pomegranate-peel-derived biochar (PG-B), for removing heavy-metal pollutants has been demonstrated. Adsorption experiments, varied characterizations, and model analyses were conducted to reveal the key role that critical experiment parameters play in the removal performance of the studied adsorbents and clarify the adsorption mechanism of Cr(VI). For the pomegranate-peel-derived biochar treated with calcination and chemical modification by Na_2_S_2_O_5_, the Cr(VI) adsorption behavior was found to follow the second-order kinetic model. For the pomegranate peel adsorption material treated by alkali, the Elovich and Langmuir isotherm models presented good applicability in describing Cr(VI) adsorption. Chemisorption and electrostatic attraction dominated the adsorption process of Cr(VI) on the two materials studied. Our study can help understand the role of pomegranate peel-derived biochar in the remediation of heavy metals and open up a new strategy to obtain high-efficiency adsorbents for environment purification.

## Figures and Tables

**Table 1:** FT-IR analysis of Cr(VI) unloaded and Cr(VI) loaded adsorbents.

Functional Group	Adsorption Peaks of PG (cm^−1^)		Adsorption Peaks of PG-B (cm^−1^)	
	Before Reaction	After Reaction	Before Reaction	After Reaction
−OH	3389	3387	3402	3422
C−H	2931	2935	2925	2929
C=O	1731	1737	—	—
C=C	1616	1640	1558	1566
C−N	1353	1366	1385	1395
C−O	1043	1063	1112	1098
C−H	—	—	956	961
N−H	—	—	625	606

**Table 2:** Parameters of Langmuir, Freundlich, and D-R models of Cr(VI) adsorption on PG and PG-B.

Material	Langmuir Isotherm			Freundlich Isotherm			D-R Model		
	Q_0_ (mg g^−1^)	K_L_ (mg L^−1^)	R^2^	K_F_ (mg g^−1^)(L mg^−1^)^1/n^	n	R^2^	Q_0_ (mmol g^−1^)	E (J mol^−1^)	R^2^
PG	12.949	0.0130	0.9859	1.226	1.533	0.9698	10.256	12.231	0.9854
PG-B	16.229	0.0114	0.9945	1.486	0.981	0.9750	12.564	13.875	0.9786

**Table 3:** Comparison of adsorption capacity of various bio-waste adsorbents for pollutants.

Pollutants	Sorbent	Operating Conditions	Adsorption Capacity (mg g^−1^)	References
Methylene blue dye	Dragon fruit peel s activated carbon	pH 10, dose 80 mg, contact time 60 min	195.2	[56]
Methylene blue dye	Kiwi peel, cucumber peel, and potato peel activated carbon	pH 6.3, dose 25 mg, contact time 180 min	435, 476, 385	[57]
Blue-106 dye	Pomegranate peel activated carbon	pH 2, dose 250 mg, contact time 120 min	58.14	[58]
Cu(II)	Raw pomegranate peel	pH 5.8, dose 250 mg, contact time 120 min	30.12	[32]
Pb(II)	Pomelo fruit peel-derived biochar	pH 5, dose 100 mg, contact time 120 min	90.3	[59]
Cu(II)	Pineapple peel	pH 5, dose 400 mg, contact time 30 min	64.33	[60]
Cr(VI)	Pomegranate peel	pH 2, dose 100 mg, contact time 120 min	38.29	[61]
Cr(VI)	Orange peel	pH 2, dose 112 mg, contact time 300 min	7.14	[62]
Cr(VI)	Modified Litchi peel	pH 4, dose 80 mg, contact time 100 min	9.55	[63]
Cr(VI)	Coconut shell	pH 2.3, dose 500 mg, contact time 200 min	8.73	[64]
Cr(VI)	Pomegranate-Peel-Derived Biochar	pH 4, dose 250 mg, contact time 30 min	16.23	This study

**Table 4:** Parameters of pseudo-first-order and pseudo-second-order kinetic models for the adsorption of Cr(VI) on PG and PG-B.

Material	Pseudo-First-Order Model			Pseudo-Second-Order Model			Elovich		
	k_1_ (min^−1^)	Q_e_ (mg g^−1^)	R^2^	k_2_ (g mg^−1^ min^−1^)	Q_e_ (mg g^−1^)	R^2^	a (g mg^−1^ min^−1^)	b (mg g^−1^ min^0.5^)	R^2^
PG	0.017	3.679	0.5584	0.148	3.813	0.8925	6.879	3.368	0.9668
PG-B	0.081	4.316	0.9397	0.102	4.489	0.9547	8.265	5.283	0.7585

## Data Availability

The data that support the findings of this study are available from the corresponding author upon reasonable request.

## References

[1] Sun W., Li J., Li H., Jin B., Li Z., Zhang T., Zhu X. (2022). Mechanistic insights into ball milling enhanced montmorillonite modification with tetramethylammonium for adsorption of gaseous toluene. Chemosphere.

[2] Wang C.C., Du X.D., Li J., Guo X.X., Wang P., Zhang J. (2016). Photocatalytic Cr(VI) reduction in metal-organic frameworks: A mini-review. Appl. Catal. B-Environ..

[3] Islam M.M., Mohana A.A., Rahman M.A., Rahman M., Naidu R., Rahman M.M. (2023). A Comprehensive Review of the Current Progress of Chromium Removal Methods from Aqueous Solution. Toxics.

[4] Das S., Mishra J., Das S.K., Pandey S., Rao D.S., Chakraborty A., Sudarshan M., Das N., Thatoi H. (2014). Investigation on mechanism of Cr(VI) reduction and removal by Bacillus amyloliquefaciens, a novel chromate tolerant bacterium isolated from chromite mine soil. Chemosphere.

[5] Yu K., Xu J., Jiang X., Liu C., McCall W., Lu J. (2017). Stabilization of heavy metals in soil using two organo-bentonites. Chemosphere.

[6] Wang D., Zhang G., Dai Z., Zhou L., Bian P., Zheng K., Wu Z., Cai D. (2018). Sandwich-like Nanosystem for Simultaneous Removal of Cr(VI) and Cd(II) from Water and Soil. ACS Appl. Mater. Interfaces.

[7] He Y., Lin H., Luo M., Liu J., Dong Y., Li B. (2020). Highly efficient remediation of groundwater co-contaminated with Cr(VI) and nitrate by using nano-Fe/Pd bimetal-loaded zeolite: Process product and interaction mechanism. Environ. Pollut..

[8] Fu L., Feng A., Xiao J., Wu Q., Ye Q., Peng S. (2021). Remediation of soil contaminated with high levels of hexavalent chromium by combined chemical-microbial reduction and stabilization. J. Hazard. Mater..

[9] Chen N., Cao S., Zhang L., Peng X., Wang X., Ai Z., Zhang L. (2021). Structural dependent Cr(VI) adsorption and reduction of biochar: Hydrochar versus pyrochar. Sci. Total Environ..

[10] Zhitkovich A. (2011). Chromium in Drinking Water: Sources, Metabolism, and Cancer Risks. Chem. Res. Toxicol..

[11] Singh V., Singh N., Rai S.N., Kumar A., Singh A.K., Singh M.P., Sahoo A., Shekhar S., Vamanu E., Mishra V. (2023). Heavy Metal Contamination in the Aquatic Ecosystem: Toxicity and Its Remediation Using Eco-Friendly Approaches. Toxics.

[12] Park J.E., Shin J.H., Oh W., Choi S.J., Kim J., Kim C., Jeon J. (2022). Removal of Hexavalent Chromium(VI) from Wastewater Using Chitosan-Coated Iron Oxide Nanocomposite Membranes. Toxics.

[13] Liang H., Zhang H., Zhao P., Zhao X., Sun H., Geng Z., She D. (2021). Synthesis of a novel three-dimensional porous carbon material and its highly selective Cr(VI) removal in wastewater. J. Clean. Prod..

[14] Xia S., Song Z., Jeyakumar P., Bolan N., Wang H. (2020). Characteristics and applications of biochar for remediating Cr(VI)-contaminated soils and wastewater. Environ. Geochem. Health.

[15] Fu F., Wang Q. (2011). Removal of heavy metal ions from wastewaters: A review. J. Environ. Manag..

[16] Li L., Cao G., Zhu R. (2021). Adsorption of Cr(VI) from aqueous solution by a litchi shell-based adsorbent. Environ. Res..

[17] Xu H., Liu Y., Liang H., Gao C., Qin J., You L., Wang R., Li J., Yang S. (2021). Adsorption of Cr(VI) from aqueous solutions using novel activated carbon spheres derived from glucose and sodium dodecylbenzene sulfonate. Sci. Total Environ..

[18] Zeng B., Xu W., Khan S.B., Wang Y., Zhang J., Yang J., Su X., Lin Z. (2021). Preparation of sludge biochar rich in carboxyl/hydroxyl groups by quenching process and its excellent adsorption performance for Cr(VI). Chemosphere.

[19] Basu M., Guha A.K., Ray L. (2017). Adsorption of Lead on Cucumber Peel. J. Clean. Prod..

[20] Huang X., Wei D., Zhang X., Fan D., Sun X., Du B., Wei Q. (2019). Synthesis of amino-functionalized magnetic aerobic granular sludge-biochar for Pb(II) removal: Adsorption performance and mechanism studies. Sci. Total Environ..

[21] Liu L., Huang Y., Zhang S., Gong Y., Su Y., Cao J., Hu H. (2019). Adsorption characteristics and mechanism of Pb(II) by agricultural waste-derived biochars produced from a pilot-scale pyrolysis system. Waste Manag..

[22] Jha S., Gaur R., Shahabuddin S., Tyagi I. (2023). Biochar as Sustainable Alternative and Green Adsorbent for the Remediation of Noxious Pollutants: A Comprehensive Review. Toxics.

[23] Alharbi H.A., Alotaibi K.D., EL-Saeid M.H., Giesy J.P. (2023). Polycyclic Aromatic Hydrocarbons (PAHs) and Metals in Diverse Biochar Products: Effect of Feedstock Type and Pyrolysis Temperature. Toxics.

[24] Olea-Mejia O., Cabral-Prieto A., Salcedo-Castillo U., Lopez-Tellez G., Olea-Cardoso O., Lopez-Castanares R. (2017). Orange peel plus nanostructured zero-valent-iron composite for the removal of hexavalent chromium in water. Appl. Surf. Sci..

[25] Rosales E., Meijide I., Tavares T., Pazos M., Sanroman M.A. (2016). Grapefruit peelings as a promising biosorbent for the removal of leather dyes and hexavalent chromium. Process. Saf. Environ. Prot..

[26] Enniya I., Rghioui L., Jourani A. (2018). Adsorption of hexavalent chromium in aqueous solution on activated carbon prepared from apple peels. Sustain. Chem. Pharm..

[27] Shakya A., Nunez-Delgado A., Agarwal T. (2019). Biochar synthesis from sweet lime peel for hexavalent chromium remediation from aqueous solution. J. Environ. Manag..

[28] Kurniawan A., Sisnandy V.O.A., Trilestari K., Sunarso J., Indraswati N., Ismadji S. (2011). Performance of durian shell waste as high capacity biosorbent for Cr(VI) removal from synthetic wastewater. Ecol. Eng..

[29] Yin Z., Xu S., Liu S., Xu S., Li J., Zhang Y. (2020). A novel magnetic biochar prepared by K2FeO4-promoted oxidative pyrolysis of pomelo peel for adsorption of hexavalent chromium. Bioresour. Technol..

[30] Rashtbari Y., Hazrati S., Azari A., Afshin S., Fazlzadeh M., Vosoughi M. (2020). A novel, eco-friendly and green synthesis of PPAC-ZnO and PPAC-nZVI nanocomposite using pomegranate peel: Cephalexin adsorption experiments, mechanisms, isotherms and kinetics. Adv. Powder Technol..

[31] Gullon B., Pintado M.E., Perez-Alvarez J.A., Viuda-Martos M. (2016). Assessment of polyphenolic profile and antibacterial activity of pomegranate peel (*Punica granatum*) flour obtained from co-product of juice extraction. Food Control..

[32] Ben-Ali S., Jaouali I., Souissi-Najar S., Ouederni A. (2017). Characterization and adsorption capacity of raw pomegranate peel biosorbent for copper removal. J. Clean. Prod..

[33] Seliem M.K., Mobarak M., Selim A.Q., Mohamed E.A., Halfaya R.A., Gomaa H.K., Anastopoulos I., Giannakoudakis D.A., Lima E.C., Bonilla-Petriciolet A. (2020). A novel multifunctional adsorbent of pomegranate peel extract and activated anthracite for Mn(VII) and Cr(VI) uptake from solutions: Experiments and theoretical treatment. J. Mol. Liq..

[34] Abdelhafez A.A., Li J.H. (2016). Removal of Pb(II) from aqueous solution by using biochars derived from sugar cane bagasse and orange peel. J. Taiwan Inst. Chem. E.

[35] Mashkoor F., Nasar A. (2019). Preparation, characterization and adsorption studies of the chemically modified Luffa aegyptica peel as a potential adsorbent for the removal of malachite green from aqueous solution. J. Mol. Liq..

[36] Waqas M., Aburiazaiza A.S., Miandad R., Rehan M., Barakat M.A., Nizami A.S. (2018). Development of biochar as fuel and catalyst in energy recovery technologies. J. Clean. Prod..

[37] Allouss D., Essamlali Y., Chakir A., Khadhar S., Zahouily M. (2020). Effective removal of Cu(II) from aqueous solution over graphene oxide encapsulated carboxymethylcellulose-alginate hydrogel microspheres: Towards real wastewater treatment plants. Environ. Sci. Pollut. Res..

[38] Huang Z., Huang Z., Feng L., Luo X., Wu P., Cui L., Mao X. (2018). Modified cellulose by polyethyleneimine and ethylenediamine with induced Cu(II) and Pb(II) adsorption potentialities. Carbohydr. Polym..

[39] Sajjadi S.-A., Meknati A., Lima E.C., Dotto G.L., Ileana Mendoza-Castillo D., Anastopoulos I., Alakhras F., Unuabonah E.I., Singh P., Hosseini-Bandegharaei A. (2019). A novel route for preparation of chemically activated carbon from pistachio wood for highly efficient Pb(II) sorption. J. Environ. Manag..

[40] Ali M.E.M., Abdelsalam H., Ammar N.S., Ibrahim H.S. (2018). Response surface methodology for optimization of the adsorption capability of ball-milled pomegranate peel for different pollutants. J. Mol. Liq..

[41] Shang J., Guo Y., He D., Qu W., Tang Y., Zhou L., Zhu R. (2021). A novel graphene oxide-dicationic ionic liquid composite for Cr(VI) adsorption from aqueous solutions. J. Hazard. Mater..

[42] Lan Huong N., Huu Tap V., Quang Trung N., Thu Huong N., Thi Bich Lien N., Van Quang N., Thu Uyen B., Hung Le S. (2021). Paper waste sludge derived-hydrochar modified by iron (III) chloride for effective removal of Cr(VI) from aqueous solution: Kinetic and isotherm studies. J. Water Process Eng..

[43] Zhou L., Liu Y., Liu S., Yin Y., Zeng G., Tan X., Hu X., Hu X., Jiang L., Ding Y. (2016). Investigation of the adsorption-reduction mechanisms of hexavalent chromium by ramie biochars of different pyrolytic temperatures. Bioresour. Technol..

[44] Fang L., Ding L., Ren W., Hu H., Huang Y., Shao P., Yang L., Shi H., Ren Z., Han K. (2021). High exposure effect of the adsorption site significantly enhanced the adsorption capacity and removal rate: A case of adsorption of hexavalent chromium by quaternary ammonium polymers (QAPs). J. Hazard. Mater..

[45] Lu M., Guan X.-H., Xu X.-H., Wei D.-Z. (2013). Characteristic and mechanism of Cr(VI) adsorption by ammonium sulfamate-bacterial cellulose in aqueous solutions. Chin. Chem. Lett..

[46] Sun Y., Yue Q., Gao B., Gao Y., Li Q., Wang Y. (2013). Adsorption of hexavalent chromium on Arundo donax Linn activated carbon amine-crosslinked copolymer. Chem. Eng. J..

[47] Albadarin A.B., Al-Muhtaseb A.a.H., Al-laqtah N.A., Walker G.M., Allen S.J., Ahmad M.N.M. (2011). Biosorption of toxic chromium from aqueous phase by lignin: Mechanism, effect of other metal ions and salts. Chem. Eng. J..

[48] Pavithra S., Thandapani G., Sugashini S., Sudha P.N., Alkhamis H.H., Alrefaei A.F., Almutairi M.H. (2021). Batch adsorption studies on surface tailored chitosan/orange peel hydrogel composite for the removal of Cr(VI) and Cu(II) ions from synthetic wastewater. Chemosphere.

[49] Touihri M., Guesmi F., Hannachi C., Hamrouni B., Sellaoui L., Badawi M., Poch J., Fiol N. (2021). Single and simultaneous adsorption of Cr(VI) and Cu (II) on a novel Fe3O4/pine cones gel beads nanocomposite: Experiments, characterization and isotherms modeling. Chem. Eng. J..

[50] Rafiaee S., Samani M.R., Toghraie D. (2020). Removal of hexavalent chromium from aqueous media using pomegranate peels modified by polymeric coatings: Effects of various composite synthesis parameters. Synth. Met..

[51] Oliveira M.R.F., Abreu K.D., Romao A.L.E., Davi D.M.B., Magalhaes C.E.D., Carrilho E.N.V.M., Alves C.R. (2021). Carnauba (Copernicia prunifera) palm tree biomass as adsorbent for Pb(II) and Cd(II) from water medium. Environ. Sci. Pollut. Res..

[52] Amin M.T., Alazba A.A., Shafiq M. (2019). Application of the biochar derived from orange peel for effective biosorption of copper and cadmium in batch studies: Isotherm models and kinetic studies. Arab. J. Geosci..

[53] Wang C., Gu L., Liu X., Zhang X., Cao L., Hu X. (2016). Sorption behavior of Cr(VI) on pineapple-peel-derived biochar and the influence of coexisting pyrene. Int. Biodeterior. Biodegrad..

[54] Hoang L.P., Van H.T., Nguyen L.H., Mac D.H., Vu T.T., Ha L.T., Nguyen X.C. (2019). Removal of Cr(VI) from aqueous solution using magnetic modified biochar derived from raw corncob. N. J. Chem..

[55] Dong F.X., Yan L., Zhou X.H., Huang S.T., Liang J.Y., Zhang W.X., Guo Z.W., Guo P.R., Qian W., Kong L.J. (2021). Simultaneous adsorption of Cr(VI) and phenol by biochar-based iron oxide composites in water: Performance, kinetics and mechanism. J. Hazard. Mater..

[56] Jawad A.H., Abdulhameed A.S., Wilson L.D., Syed-Hassan SS A., Alothman Z.A., Khan M.R. (2021). High surface area and mesoporous activated carbon from KOH-activated dragon fruit peels for methylene blue dye adsorption: Optimization and mechanism study. Chin. J. Chem. Eng..

[57] Mahmoodi N.M., Taghizadeh M., Taghizadeh A. (2018). Mesoporous activated carbons of low-cost agricultural bio-wastes with high adsorption capacity: Preparation and artificial neural network modeling of dye removal from single and multicomponent (binary and ternary) systems. J. Mol. Liq..

[58] Amin N.K. (2009). Removal of direct blue-106 dye from aqueous solution using new activated carbons developed from pomegranate peel: Adsorption equilibrium and kinetics. J. Hazard. Mater..

[59] Dinh V.-P., Nguyen D.-K., Luu T.-T., Nguyen Q.-H., Tuyen L.A., Phong D.D., Kiet HA T., Ho T.-H., Nguyen TT P., Xuan T.D. (2022). Adsorption of Pb(II) from aqueous solution by pomelo fruit peel-derived biochar. Mater. Chem. Phys..

[60] Romero-Cano L.A., Garcia-Rosero H., Gonzalez-Gutierrez L.V., Baldenegro-Perez L.A., Carrasco-Marin F. (2017). Functionalized adsorbents prepared from fruit peels: Equilibrium, kinetic and thermodynamic studies for copper adsorption in aqueous solution. J. Clean. Prod..

[61] Giri R., Kumari N., Behera M., Sharma A., Kumar S., Kumar N., Singh R. (2021). Adsorption of hexavalent chromium from aqueous solution using pomegranate peel as low-cost biosorbent. Environ. Sustain..

[62] Ben Khalifa E., Rzig B., Chakroun R., Nouagui H., Hamrouni B. (2019). Application of response surface methodology for chromium removal by adsorption on low-cost biosorbent. Chemom. Intell. Lab. Syst..

[63] Yi Y., Lv J., Liu Y., Wu G. (2017). Synthesis and application of modified Litchi peel for removal of hexavalent chromium from aqueous solutions. J. Mol. Liq..

[64] Nag S., Mondal A., Bar N., Das S.K. (2017). Biosorption of chromium (VI) from aqueous solutions and ANN modelling. Environ. Sci. Pollut. Res..

[65] Wang X.S., Chen L.F., Li F.Y., Chen K.L., Wan W.Y., Tang Y.J. (2010). Removal of Cr (VI) with wheat-residue derived black carbon: Reaction mechanism and adsorption performance. J. Hazard. Mater..

[66] Jain M., Garg V.K., Kadirvelu K. (2010). Adsorption of hexavalent chromium from aqueous medium onto carbonaceous adsorbents prepared from waste biomass. J. Environ. Manag..

[67] Bansal M., Singh D., Garg V.K. (2009). A comparative study for the removal of hexavalent chromium from aqueous solution by agriculture wastes’ carbons. J. Hazard. Mater..

[68] Dobrowolski R., Otto M. (2010). Study of chromium(VI) adsorption onto modified activated carbons with respect to analytical application. Adsorption.

[69] Qin L., He L., Yang W., Lin A. (2020). Preparation of a novel iron-based biochar composite for removal of hexavalent chromium in water. Environ. Sci. Pollut. Res..

[70] Shi J., Simal-Gandara J., Mei J., Ma W., Peng Q., Shi Y., Xu Q., Lin Z., Lv H. (2021). Insight into the pigmented anthocyanins and the major potential co-pigmented flavonoids in purple-coloured leaf teas. Food Chem..

[71] Sun X., Li C., Ma J., Zang Y., Huang J., Chen N., Wang X., Zhang D. (2021). New amide alkaloids and carbazole alkaloid from the stems of Clausena lansium. Fitoterapia.

[72] Mahindrakar K.V., Rathod V.K. (2020). Ultrasonic assisted aqueous extraction of catechin and gallic acid from Syzygium cumini seed kernel and evaluation of total phenolic, flavonoid contents and antioxidant activity. Chem. Eng. Process..

[73] Shahkoomahally S., Khadivi A., Brecht J.K., Sarkhosh A. (2021). Chemical and physical attributes of fruit juice and peel of pomegranate genotypes grown in Florida, USA. Food Chem..

[74] Jia X., Zhang Y., He Z., Chang F., Zhang H., Wagberg T., Hu G. (2021). Mesopore-rich badam-shell biochar for efficient adsorption of Cr(VI) from aqueous solution. J. Environ. Chem. Eng..

[75] Ranasinghe S.H., Navaratne A.N., Priyantha N. (2018). Enhancement of adsorption characteristics of Cr(III) and Ni(II) by surface modification of jackfruit peel biosorbent. J. Environ. Chem. Eng..

